# Potential link between tastes preference and digestive system cancer hospitalisations in Fujian Province, China: big data analytics

**DOI:** 10.1017/S1368980025101419

**Published:** 2025-11-06

**Authors:** Zhixiang Rao, Jingru Huang, Zhaocheng Zhuang, Jing Zheng, Xuwei Tang, Hanwei Wang, Zhijian Hu, Xiane Peng

**Affiliations:** 1 School of Humanities and Management, Fujian University of Traditional Chinese Medicine, Fuzhou 350122, China; 2 School of Integrated Chinese and Western medicine, Fujian University of Traditional Chinese Medicine, Fuzhou 350122, China; 3 Department of Epidemiology and Health Statistics, School of Public Health, Quanzhou Medical College, Quanzhou, Fujian 362011, China; 4 Department of Epidemiology and Health Statistics, Fujian Provincial Key Laboratory of Environment Factors and Cancer, School of Public Health, Fujian Medical University, Fuzhou 350122, China; 5 Infectious Disease Prevention and Control Department, Fuzhou Center for Disease Control and Prevention, Fuzhou 350004, China; 6 Key Laboratory of Ministry of Education for Gastrointestinal Cancer, Fujian Medical University, Fuzhou 350122, China

**Keywords:** Taste preference index, Digestive system cancer hospitalisation rates, Internet big data, Spatial aggregation, GeoDetector

## Abstract

**Objective:**

The study aimed to utilise internet big data to quantify the taste preferences of residents in Fujian Province and to explore the relationship between dietary taste preferences and hospitalisation rates for digestive system cancers.

**Design:**

The study employed an associative design using internet big data to analyse dietary behaviour and its association with hospitalisation rates for digestive system cancers. GeoDetector methods were used to compare the association between rural residents’ hospitalisation rates and their taste preferences.

**Setting:**

This study utilised internet recipe data to collect cuisines taste information. By integrating this with categorised restaurant data from point of interest sources across various regions in Fujian province, it quantitatively analysed the regional taste preferences of people.

**Participants:**

Data from seventy-two counties in Fujian cover most of the province. Included 154 686 hospitalisation records for digestive system cancers (2010–2016) from the New Rural Cooperative Medical Scheme database, 16 363 recipes from Internet and data from 30 984 restaurants through Amap.

**Results:**

The study found pungent to be the prevalent taste in Fujian, with salty, spicy and sour following. Coastal areas favoured stronger tastes. Spatial analysis showed taste preferences clustered geographically, with Sour and Fat tastes having an association with liver and colorectal cancer (CC) hospitalisations, though with modest association values (0·110–0·199).

**Conclusions:**

The study found significant spatial clustering of taste preferences in Fujian Province and an association between Sour and Fat tastes preference and hospitalisation rates for liver and CC, suggesting a dietary taste–cancer link.

Digestive system cancer is the most common and lethal cancer in the world, mainly including gastric cancer (GC), colorectal cancer (CRC), oesophageal cancer (EC), liver cancer (LC), and pancreatic cancer (PC)^(1)^. Digestive system cancers are among the most common types of cancers worldwide, and their high incidence and mortality rates pose a significant threat to public health^(2)^. Based on the global cancer statistics, China’s cancer mortality rate is 30 % and 40 % higher than those of the United Kingdom and the United States, respectively. Notably, gastrointestinal cancers, including gastric, liver and oesophageal cancers, contribute to 36·4 % of all cancer-related deaths in China. In contrast, in the United States and the United Kingdom, such cancers account for no more than 5 % of total cancer mortality^(3)^. The prevalence of digestive system cancers is impacting the health of the Chinese population.

The occurrence of digestive system cancers is closely related to a variety of behavioural and lifestyle factors, which have both commonalities and unique characteristics. Understanding the role of these behavioural factors is crucial for developing effective prevention strategies. One-third of cancer risk is associated with smoking, drinking, poor diet and lack of exercise, behavioural factors that significantly impact cancer hospitalisation rates. In 2017, sub-optimal diets caused 11 million deaths globally^(4,5)^. Dietary behaviour is an important factor affecting the occurrence and development of cancer, especially digestive system cancers^(6,7)^. For example, the Western dietary pattern (high in fat, protein and red meat) increases the proportion of opportunistic pathogens in the gut while reducing the proportion of symbiotic bacteria, thereby promoting the development of CC^(8)^. Research has also found that dietary behaviour not only affects the risk of cancer development but may also influence the prognosis of cancer patients. For instance, one study found that among CC patients, continued high consumption of red and processed meat after diagnosis was associated with a significantly elevated risk of overall mortality, particularly in those harboring KRAS mutations^(9)^. Moreover, chemotherapy and radiotherapy may lead to changes in taste perception, which in turn can affect cancer patients’ enjoyment of food and dietary intake^(10,11)^. Despite research on healthy eating indicating that differences in dietary choices among various genders, dietary patterns and types of food lead to different health outcomes^(12–16)^, research investigating the association between the extent of taste preference alterations and corresponding changes in health status remain limited in current literature. In summary, from a public health perspective, the role of taste preference alterations in cancer incidence and progression requires further investigation to develop more effective population dietary behaviour interventions and lifestyle guidance strategies.

Traditional dietary behaviour surveys are often time consuming and expensive. In recent years, Internet big data has been widely applied across numerous industries. Internet big data is characterised by its extensive coverage, strong objectivity, large data volume, reliability, cost-effectiveness and accuracy^(17–19)^. We aimed to quantify the taste preferences of residents in Fujian Province by internet big data and explored the relationship between dietary taste and hospitalisation for digestive system cancer. The use of internet big data offers a complementary approach to traditional dietary behaviour surveys, enhancing objectivity and efficiency. By leveraging large-scale data, we can gain a comprehensive understanding of taste preferences, which supports more robust research outcomes.

Fujian, as the birthplace of Min cuisine – one of the eight major culinary traditions in China – has developed due to its long-standing culinary history. As a result, residents in the Fujian region are more particular about cooking dishes and often add a greater variety of condiments^(20)^. Additionally, the regional dietary culture of Fujian is influenced by traditional Chinese medicinal diets, which combine herbs with food to prevent and treat diseases or improve health^(21)^. Such as the ‘Kejia’ (Hakka) communities in Fujian, where medicinal plants are traditionally used to make soups to promote health and prevent illness^(22)^. Notably, in Fujian diets, certain condiments with medicinal properties are also commonly used, which aligns with the traditional Chinese medical theory that food and medicine share the same origin^(23)^. Based on this, this study selects the Fujian province as the research area to conduct a survey on residents’ taste preferences, with the expectation of providing new research insights for cancer prevention and control in the population.

## Methods

### Data source

#### Research area and data

The geographical scope of this study is confined to Fujian Province. Fujian, abbreviated as ‘Min,’ is located on the southeast coast of China, facing Taiwan across the sea. The province spans 530 kilometers from north to south and 480 kilometers from east to west, covering nine prefecture-level cities: Fuzhou, Xiamen, Putian, Quanzhou, Zhangzhou, Longyan, Sanming, Nanping and Ningde. Given the limitations of the data in this study, it only covers the vast majority of counties in Fujian Province. Ultimately, data from seventy-two counties were included in this study.

#### Data of common digestive system cancer hospitalisation rates

Our data originates from the government-administered medical insurance department’s New Rural Cooperative Medical Scheme database, and we have obtained authorisation from the relevant authorities. From 2010 to 2016, we collected hospitalisation records for common digestive system cancers from counties across Fujian Province. These records were classified according to the International Classification of Diseases, 10th Edition (ICD-10). Specifically, the data includes four types of digestive system cancers: oesophageal cancer (EC, C15), gastric cancer (GC, C16), CC (CRC, C18-21) and liver cancer (LC, C22). Recent studies have indicated that China has largely achieved comprehensive medical insurance coverage, with the participation rate of rural residents in the New Rural Cooperative Medical Scheme reaching 96·6 % in 2010^(24)^.

The hospitalisation record information we used includes patient hospitalisation ID numbers, disease diagnosis, residential addresses, gender, age, admission and discharge dates, etc. Due to the recurrent nature and particularities of cancer, there may be cases of repeated hospitalisations for cancer patients. For the purpose of this study, only the data from the first hospitalisation were recorded. To protect patient privacy, identifiable information has been anonymised. Population data were sourced from the Fujian Provincial Statistical Yearbook (http://tjj.fujian.gov.cn/xxgk/ndsj/). After excluding erroneous and unidentifiable data, we ultimately included a total of 154 686 hospitalisation records covering the period from 2010 to 2016.

#### Website recipe data

We conducted a search using the keyword ‘recipes’ in search engines and selected ‘Meishichina’ (https://www.meishichina.com/) as the data source website based on search engine rankings, the ease of web data crawling, the diversity of cuisine classification, the number of recipes and whether condiments are used. Based on the source page, we obtained the materials of the recipes, including main ingredients, auxiliary ingredients and condiments. We obtained a total of 16 363 recipes for the twenty-five types of cuisines. The types of recipes are shown in online supplementary material, Supplemental Table 1.

#### Data of points of interests of restaurant of Fujian

The points of interest of restaurants in various regions of the Fujian Research Area were obtained through Amap (https://www.amap.com/). Amap is China’s leading provider of digital map content, navigation and location-based service solutions. Amap offers a wide range of location search and surrounding services, including restaurants, hotels and gas stations. Based on the points of interest data tags provided by the Amap Developer Platform, we crawled the names and coordinates of restaurants in all districts and counties and classified the cuisines they serve according to the data tags. Since Chinese cuisine often involves the use of condiments, we selected only Chinese restaurants from the points of interest data tags. In the end, we obtained a total of 30 984 Chinese restaurants across twenty-two categories through Amap.

### The global index of spatial autocorrelation – Moran’s I

To determine whether regions with similar quantities of each component are clustered or randomly distributed across the study area, we use the Moran’s I index to explore this outcome^(25)^.

The global index of spatial autocorrelation can be identified using the following equation^(26,27)^.
(1)

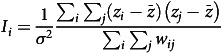




where 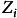 is the value of the variable *Z* at the location *i*, *Z* is the average value of *Z* with the sample number of *n*, 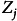 is the value of the variable *Z* at all the other locations (where *j* ≠ *i*), 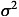 is the variance of variable *Z* and 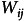 is a weight which can be defined as the inverse of the distance ( 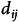 ) or the inverse of the distance squared ( 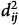 ) among locations *i* and *j*. And like any inferential statistic, we have to determine statistical significance before we can read the result. This is done with a simple hypothesis test, calculating a z-score and its associated *P* value.

A high value of Moran’s I and a corresponding z-score greater than 1·96 indicate that there is statistically significant clustering among the regions (*P* < 0·05). Low values of Moran’s I and z-scores less than –1·96 suggest a statistically significant regular distribution. However, the global index of spatial autocorrelation measures the overall spatial autocorrelation of the dataset but does not identify the specific locations where clusters of high or low taste indices occur. Therefore, local indicators of spatial autocorrelation are required.

### Local indicators of spatial association – Anselin local Moran’s I

Methods for identifying spatial clusters or hot spots are used in a variety of disciplines (e.g. ecology, epidemiology and geography)^(25,26,28)^.

The local Moran’s I is a relative measure and can only be interpreted within the context of its computed z-score or *P* value. They can be identified using the local Moran’s I^(26,29)^.
(2)

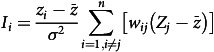




where 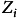 is the value of the variable *Z* at location *i*, *Z* is the average value of *Z* with the sample number of *n*, 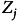 is the value of the variable *Z* at all the other locations (where *j* ≠ *i*), 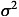 is the variance of variable *Z* and 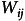 is a weight that can be defined as the inverse of the distance 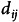 or the inverse of the distance squared 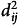 among locations *i* and *j*.

This analysis was conducted across the seventy-two counties in Fujian Province to identify groups of regions with similar taste index values that were heterogeneous within each cluster. The results of the Local Moran’s I were visualised on a map, where clusters and their values were intuitively identified by eye and classified according to cluster/outlier type. The counties were categorised into four groups: high–high (H-H) cluster, low–high (L-H) outlier, low–low (L-L) cluster and high–low (H-L) outlier. The interpretations of these groups were as follows:

High–high (H-H) cluster: Indicates that the region has a high taste index value and is surrounded by neighbouring regions with similarly high taste index values, forming a high-value cluster.

Low–high (L-H) cluster: Indicates that the region has a low taste index value but is surrounded by neighbouring regions with high taste index values, forming a low-value outlier.

Low–low (L-L) cluster: Indicates that the region has a low taste index value and is surrounded by neighbouring regions with similarly low taste index values, forming a low-value cluster.

High–low (H-L) cluster: Indicates that the region has a high taste index value but is surrounded by neighbouring regions with low taste index values, forming a high-value outlier.

### Extraction of cuisine taste

Due to the fact that recipes often contain vague descriptions of the amounts of seasonings used (such as ‘a pinch of ’, ‘a small amount of’, ‘an appropriate amount of’ and ‘a few drops of ’), it is difficult to measure the quantities of seasonings in recipes. Therefore, our study focused on quantifying the frequency of seasoning usage to quantify the taste. Referring to the research of Li, we categorised taste into seven dimensions: ‘Sour’, ‘Sweet’, ‘Umami’, ‘Salty’, ‘Fat’, ‘Spicy’ and ‘Pungent’^(16)^. Here, ‘umami’ refers to the use of tastes-enhancing condiments, such as fish sauce and oyster sauce; ‘Fat’ refers to the use of fat-related seasonings, such as lard and peanut oil; ‘spicy’ refers to the use of chili-related condiments; and ‘pungent’ refers to condiments other than chili, such as garlic, Sichuan peppercorn and ginger. Each ingredient or condiments was labelled with one or more tastes. That is to say, each time a condiment is used in recipe 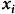 , the frequency of the corresponding taste 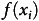 would increase by 1. Finally, we calculated the average frequency of taste for each dish.

### Construction of Taste Index Matrix

Since we obtained recipes for twenty-five types of Chinese cuisines, while there are only twenty-two corresponding categories of Chinese restaurants, it is not possible to match the recipe styles one-to-one with the restaurant categories. Therefore, it is necessary to adjust the weights of the recipes according to the restaurant categories to achieve the corresponding assignment matching. Our survey results show that Chinese restaurants, comprehensive restaurants and seafood restaurants all have similar types of recipes. As a result, this study assigned the mean value of the taste frequency of the twenty-five recipes to these three types of restaurants. Local specialty restaurants correspond to the taste frequency value of Fujian cuisine; Jiangsu cuisine restaurants often provide both Jiangsu and Huaiyang cuisines, so we calculated the average value of the taste frequencies of these two cuisines for the matching; Xibei Restaurant and Halal Restaurant often provide dishes related to Xibei and Xinjiang cuisines, so we used the average value of the taste frequencies of these two cuisines; Chaozhou cuisine restaurants offer Guangdong cuisine, so they were assigned the taste frequency index of Guangdong cuisine (Cantonese cuisine). Hot pot restaurants were assigned the taste frequency of Sichuan cuisine. The matching table between restaurants and cuisines is shown in online supplementary material, Supplemental Table 1.

Total taste preference index 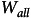 is calculated as follows:
(3)

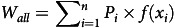




where 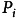 represented the proportion of each type of restaurants in the region *i*, and 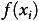 represented the taste frequency corresponding to the region *i.* The total taste preference index 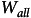 was equal to the sum of taste preference indexes of each county.

### GeoDetector

The GeoDetector (https://www.geodetector.cn/) is a statistical method used to measure the spatial stratified heterogeneity of geographical entities and to reveal the factors behind this heterogeneity^(30)^. Initially developed to assess environmental risk factors for endemic diseases, and now the GeoDetector method has since been widely applied in various fields, including social science, environmental science and human health. In this study, we introduce the GeoDetector method to explore the relationship between dietary taste and the incidence of digestive system cancers in various counties of Fujian Province.

The GeoDetector comprises three detectors:

1. The factor detector identifies the factors that cause hospitalisation risks and determines the power of these risk factors on hospitalisation rates, which is expressed by the q-value.

2. The interaction detector tests whether multiple risk factors interact with one another or affect hospitalisation rates independently by comparing the individual q-value of each factor with the q-value obtained when the factors are considered together, thereby determining whether their joint effect modifies the explanatory power for the dependent variable.

3. The risk detector indicates whether there are significant differences in hospitalisation rates between sub-regions with different taste levels.

The formula was as follows:
(4)

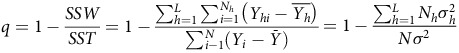




*SSW* represents the sum of variance of hospitalisation rate of each county in the sub-region divided by taste preference index level; *SST* represents the total variance of hospitalisation rate in all counties; *N* represents the total number of districts, counties and cities; 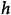 = 1, ……, L represent the level value of taste preference index after discretisation; 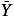 and 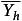 represent the average cancer hospitalisation rate of each county and the average cancer hospitalisation rate in the corresponding sub-region when the taste preference index is at the level 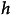 ; 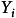 and 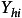 represent the cancer hospitalisation rate of county *i* and the cancer hospitalisation rate of the county *i* in the corresponding sub-region when the taste preference index is at the level 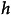 , respectively; 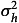 and 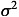 represent the variance of the cancer hospitalisation rate in the sub-region corresponding to the level *h* of the taste preference index and the variance of the cancer hospitalisation rate in all counties. Where the value range of q value was [0, 1], and q = 1 mean that the spatial distribution of the influencing factor fully explains the spatial pattern of cancer hospitalisation rate, while q = 0 mean that the spatial distribution of hospitalisation rate is completely random in space.

The GeoDetector method has advantages over traditional statistical methods, and its q statistic can avoid the interference of confounding factors that may be caused by spatial stratified heterogeneity^(31,32)^.

## Results

### Demographic characteristics

The basic characteristics of hospitalisations for four types of digestive system cancers among rural residents in Fujian Province from 1 January 2010, to 31 December 2016, were presented in Table 1. The data in this study encompassed 154 686 hospitalisation records for four common types of digestive system cancers. Stratified analysis by gender indicated that the number of male patients admitted exceeded that of female patients across all cancer types. Specifically, the proportions of male patients with oesophageal cancer, GC, liver cancer and CC were 69·83 %, 71·60 %, 79·89 % and 56·01 %, respectively. When stratified by age, the results showed that the majority of hospitalisations were among middle-aged and elderly individuals. For oesophageal cancer, GC and CC, the highest proportion of patients was in the 60+ age group. In contrast, for liver cancer, the highest proportion of patients was in the 0–50 age group.

**Table 1.** Demographic characteristics (*n* 154 686)

Cancer type	Variable	Number	Percentage (%)
Oesophageal cancer		40 628	
Sex	Male	28 370	69·83
	Female	12 258	30·17
Age	0∼	4928	12·13
	50∼	12 182	29·98
	60∼	13 151	32·37
	70∼	7684	18·91
	80∼	2683	6·60
Gastric cancer		45 671	
Sex	Male	32 702	71·60
	Female	12 969	28·40
Age	0∼	6147	13·46
	50∼	11 579	25·35
	60∼	15 367	33·65
	70∼	9929	21·74
	80∼	2649	5·80
Liver cancer		39 753	
Sex	Male	31 758	79·89
	Female	7995	20·11
Age	0∼	12 043	30·29
	50∼	11 805	29·70
	60∼	9648	24·27
	70∼	4938	12·42
	80∼	1319	3·32
Colorectal cancer		28 634	
Sex	Male	16 038	56·01
	Female	12 596	43·99
Age	0∼	6672	23·30
	50∼	7265	25·37
	60∼	8109	28·32
	70∼	4967	17·35
	80∼	1621	5·66

### Taste preference index matrix of counties in Fujian Province

#### Taste preference index of cuisine

For each cuisine, we calculated the frequency of seven tastes, and the number of recipes and the taste frequency index are shown in Table 2. For the majority of recipes, ‘Pungent’ is the most frequently used taste, followed by ‘Salty’, ‘Spicy’ and ‘Sour.’ In Guizhou cuisine, the frequency of ‘Sour’ ingredients or condiments in each dish is 0·52. In Hong Kong cuisine, the usage of sugar and fat is the highest, with each dish containing 1 unit of ‘Sweet’ taste and 0·88 unit of ‘Fat’ taste. The usage frequency of ‘Umami’ in Dongbei cuisine is the highest among all cuisines; Xinjiang cuisine has the highest frequency of ‘Pungent.’ As a local cuisine, Fujian cuisine has a relatively light taste, especially in the use of ‘Fat,’ ‘Spicy’ and ‘Pungent’ tastes, ranking lower among the twenty-five cuisines.

**Table 2.** Number and taste frequency index of recipes

Type of cuisine	Number of recipes	Sour	Sweet	Umami	Salty	Fat	Spicy	Pungent
Fujian	582	0·13	0·52	1·02	1·51	0·29	0·3	1·99
Xinjiang	322	0·1	0·45	1·25	2·01	0·32	1·4	3·93
Anhui	352	0·08	0·31	0·64	0·95	0·17	0·38	2·01
Jiangsu	100	0·13	0·5	0·73	1·01	0·25	0·16	1·77
Zhejiang	698	0·2	0·59	1·29	1·79	0·38	0·33	2·13
Guangdong	2753	0·19	0·71	1·1	1·63	0·38	0·27	1·73
Dongbei	1095	0·33	0·51	1·47	2·17	0·34	0·71	2·75
Xibei	452	0·24	0·35	1·17	1·84	0·4	1·06	3·15
Huibei	271	0·13	0·31	0·84	1·29	0·26	0·59	2·1
Shandong	1702	0·13	0·37	0·78	1·17	0·23	0·47	2·12
Hunan	832	0·25	0·42	1·34	1·89	0·36	1·33	2·82
Taiwan	425	0·2	0·91	1·01	1·51	0·47	0·26	2·13
Shanghai	88	0·11	0·45	0·92	1·23	0·24	0·27	2·06
Beijing	899	0·17	0·8	0·99	1·72	0·46	0·43	2·14
Sichuan	3429	0·35	0·5	1·46	2·22	0·47	2·08	3·38
Yunnan	168	0·14	0·33	0·62	0·98	0·27	0·6	1·48
Guizhou	112	0·52	0·12	0·53	1·02	0·2	1·13	1·88
Huaiyang	496	0·15	0·63	1·23	1·77	0·31	0·25	2·28
Jiangxi	230	0·22	0·6	1·4	1·87	0·4	0·65	2·06
Shanxi	197	0·31	0·54	1·25	2·02	0·45	0·92	2·61
Henan	215	0·09	0·24	0·52	0·98	0·31	0·33	1·8
Kejia	285	0·18	0·53	1·5	2·11	0·29	0·24	2·05
Macao	26	0	0·62	0·08	0·5	0·77	0	0·81
Hongkong	121	0·05	1	0·33	0·83	0·88	0·13	0·73
Others	513	0·12	0·32	0·35	0·6	0·24	0·65	1·86

#### Taste index matrix

**Figure 1.**
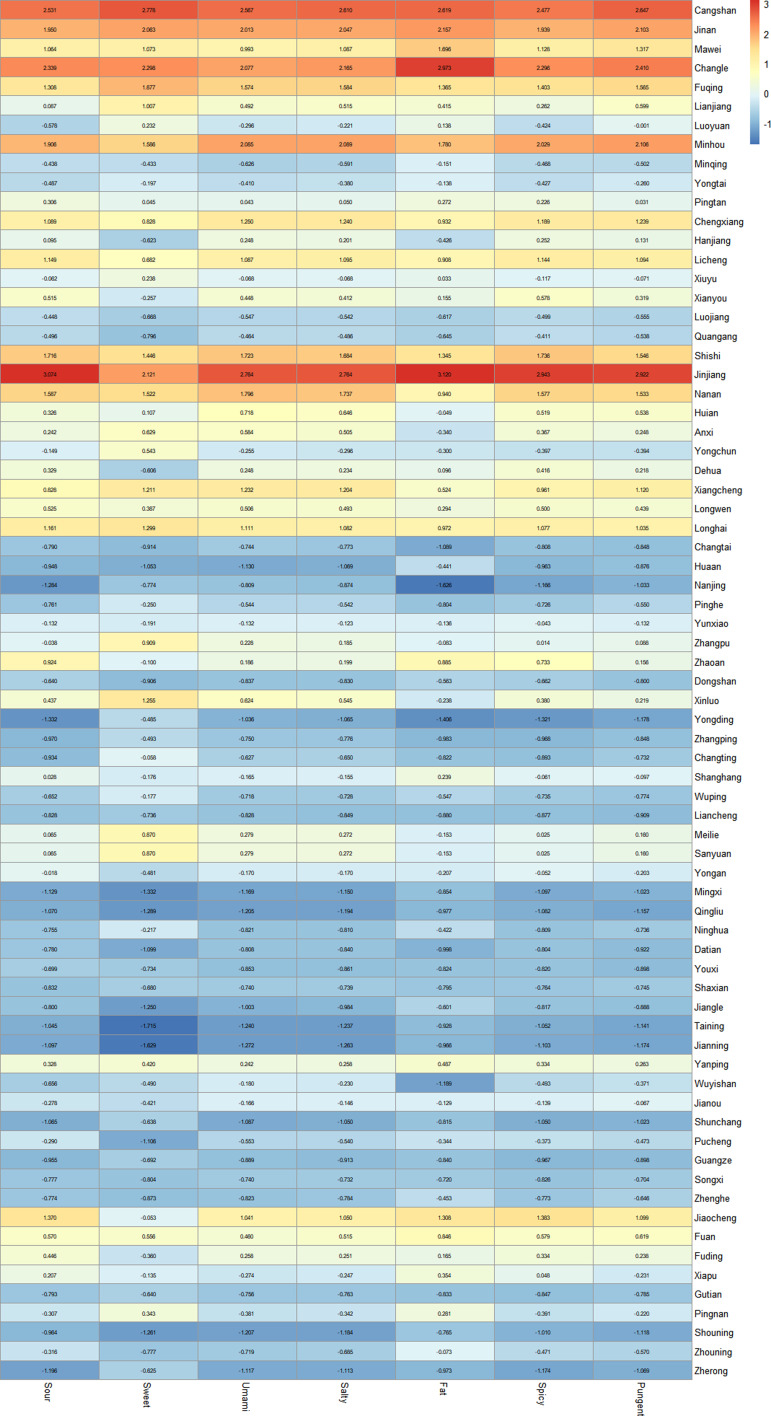
Heatmap of standardised taste index matrix of Fujian.

The points of interest data obtained from the Amap Developer API was classified according to its data tags. A total of 28 226 Chinese restaurants across twenty-two categories were obtained in seventy-two counties. In each county, the number of Chinese restaurants is the highest, followed by Fujian cuisine restaurants (including local specialties and Fujian cuisine). Based on the correspondence in online supplementary material, Supplemental Table 2, the proportion of twenty-five types of restaurants was matched with the taste frequency in the website menu to calculate the taste index for seven tastes in the seventy-two counties of Fujian Province. Then A standardised taste index matrix is constructed in Figure 1.

**Figure 2.**
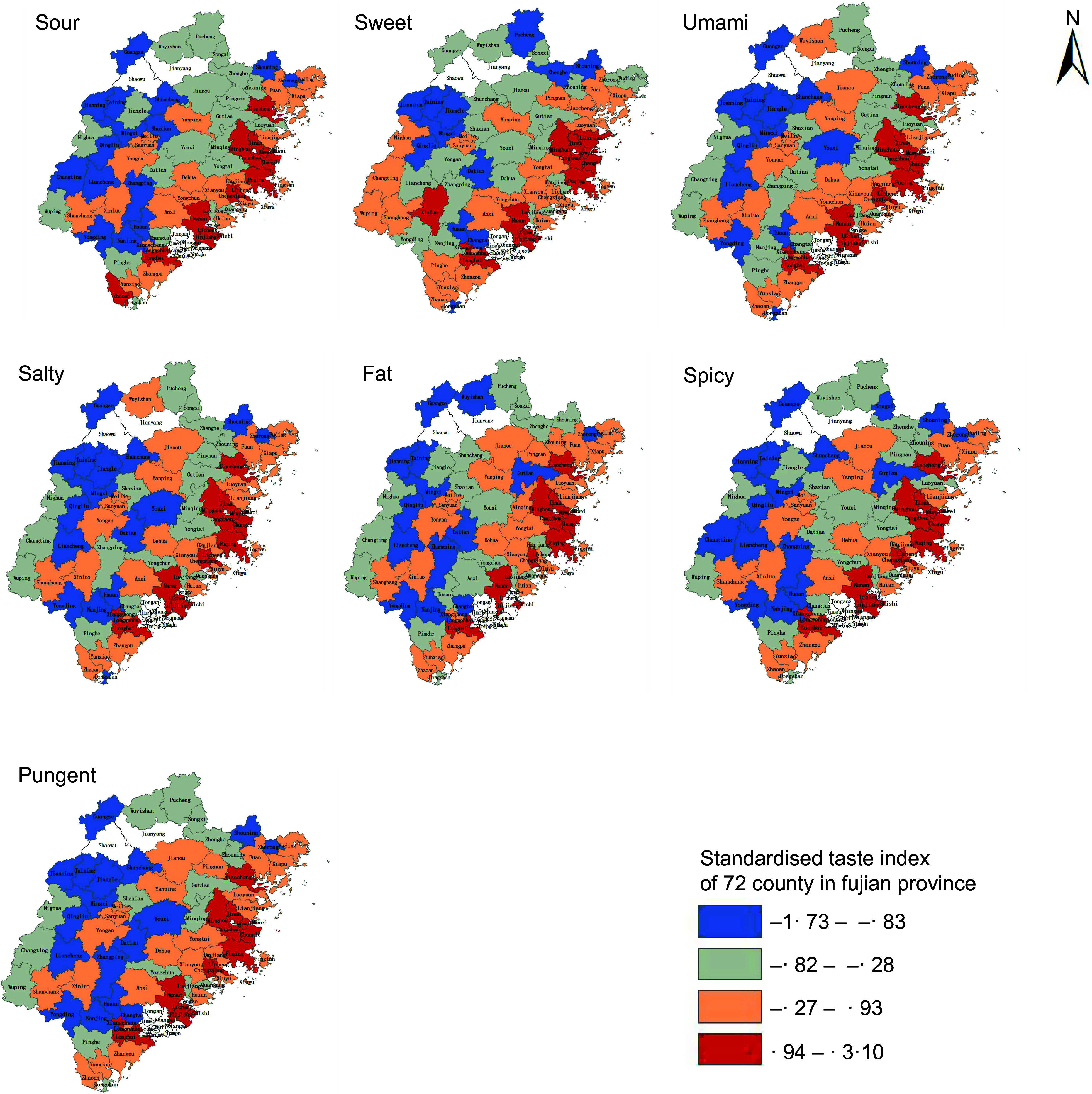
Distribution of seven kinds of taste index maps in all districts and counties of Fujian Province.

In terms of spatial distribution, the seven taste indexes in most coastal counties are higher than those in inland counties, indicating that the dietary tastes in the coastal areas of Fujian Province are heavier in Figure 2. From the city level, Fuzhou and Quanzhou have heavier tastes, while Sanming and Nanping have lighter tastes.

### Spatial aggregation of taste index

**Figure 3.**
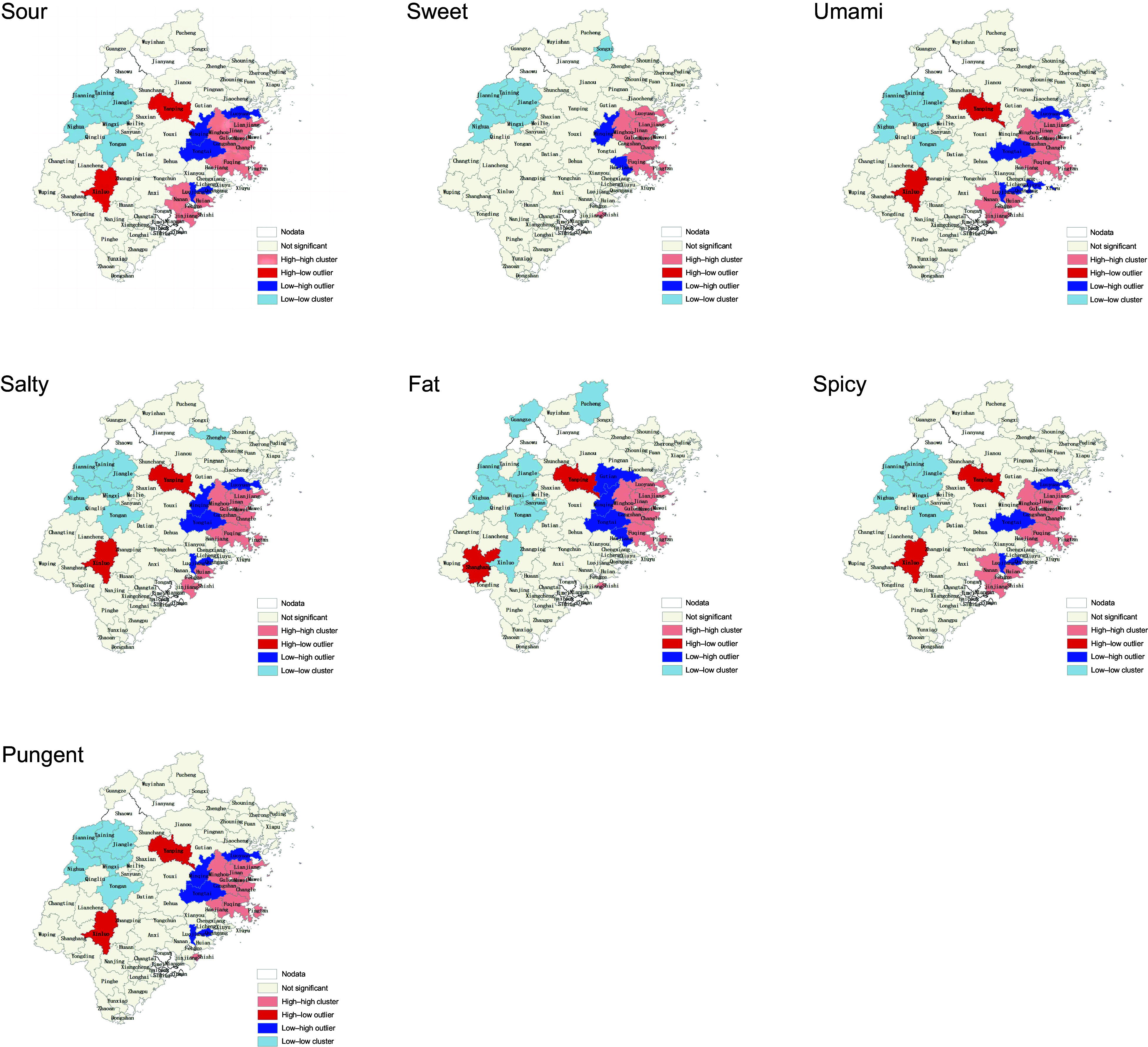
The local clustering evaluation index (LISA) clustering results. LISA, local indicators of spatial autocorrelation.

In Fujian Province, Moran’s I index results show that the seven taste indexes present an agglomeration effect in space for every district, county and city, as shown in Table 3. Spatial clustering was also observed in the local clustering evaluation index clustering results, as shown in Figure 3.

**Table 3.** Overall Moran’s I index of various taste index in Fujian province

Variable	Sour	Sweet	Umami	Salty	Fat	Spicy	Pungent
Moran’s I	0·373	0·386	0·410	0·417	0·424	0·396	0·437
*P* value	< 0·001	< 0·001	< 0·001	< 0·001	< 0·001	< 0·001	< 0·001

### Results of factor detector

#### Results of factor detector

Results as shown in Table 4 and Table 5 Sour and Fat tastes were associated with liver and CC hospitalisation rates. Results for oesophageal cancer and GC are presented in online supplementary material, Supplemental Table 3 and Table 4, showing no significant association. However, the association degree value of risk (q value) is small, with a minimum value of 0·110 and a maximum value of 0·199. Neither taste factors nor the use of Sour and Fat tastes accounted for a great part of the risk factors affecting liver cancer and CC hospitalisation rates. Additionally, the variation in the risk factors value with increasing years is also relatively small, indicating that ‘sour’ and ‘fat’ as taste risk factors are stable and long term.

**Table 4.** Risk value between liver cancer hospitalisation rate and taste index (q value)

Year	Sour	Sweet	Umami	Salty	Fat	Spicy	Pungent
2010	0·124*	0·068	0·055	0·060	0·120	0·091	0·037
2011	0·089	0·078	0·036	0·058	0·131	0·056	0·050
2012	0·125	0·073	0·054	0·076	0·150*	0·075	0·070
2013	0·115*	0·052	0·054	0·071	0·199*	0·075	0·058
2014	0·101	0·046	0·034	0·039	0·171*	0·054	0·039
2015	0·066	0·018	0·029	0·022	0·099	0·053	0·014
2016	0·040	0·039	0·016	0·012	0·099	0·033	0·005
Mean	0·103	0·051	0·039	0·046	0·159*	0·065	0·035

‘*’ represents *P* < 0·05.

**Table 5.** Risk value between colorectal cancer hospitalisation rate and taste index (q value)

Year	Sour	Sweet	Umami	Salty	Fat	Spicy	Pungent
2010	0·114	0·004	0·007	0·029	0·087	0·015	0·022
2011	0·098	0·043	0·020	0·039	0·074	0·038	0·038
2012	0·134*	0·040	0·015	0·024	0·054	0·008	0·023
2013	0·110*	0·018	0·019	0·041	0·154	0·052	0·040
2014	0·095	0·032	0·030	0·042	0·153*	0·085	0·046
2015	0·126*	0·045	0·038	0·019	0·152	0·057	0·017
2016	0·137*	0·037	0·049	0·036	0·147	0·073	0·042
Mean	0·134*	0·031	0·018	0·030	0·144*	0·048	0·032

‘*’ represents *P* < 0·05.

#### Results of risk detector

**Figure 4.**
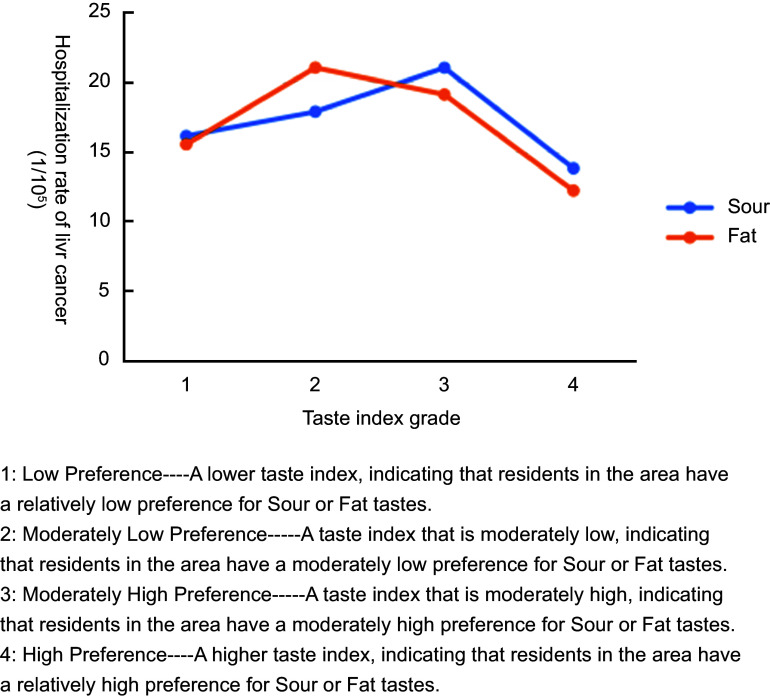
Risk Detector Results on the association of taste index grades (Sour and Fat) with Liver cancer hospitalisation rates.

**Figure 5.**
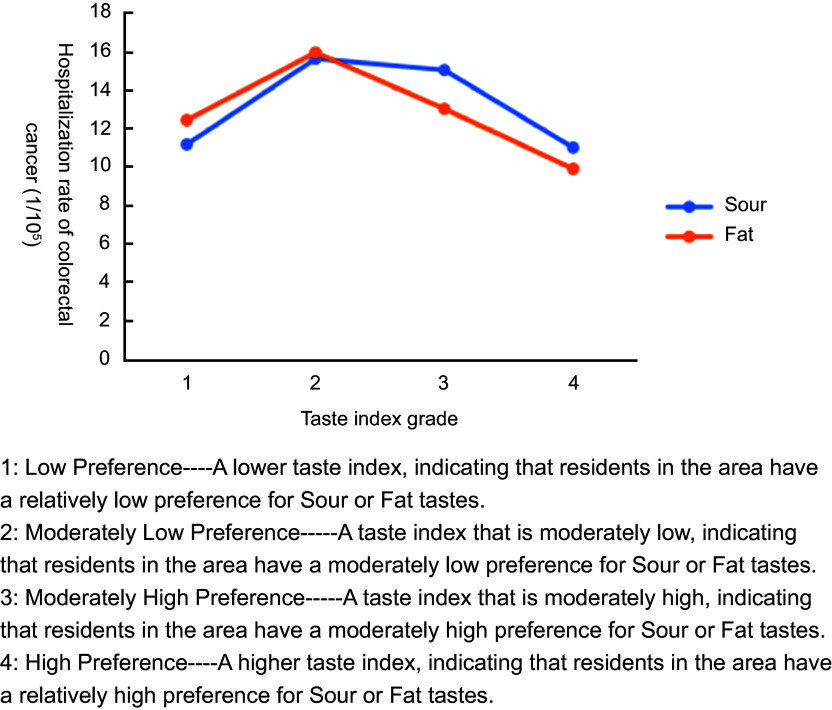
Risk detector results on the association of taste index grades (Sour and Fat) with colorectal cancer hospitalisation rates.

The results of the risk detector analysis of mean cancer hospitalisation rates from 2010 to 2016 revealed statistically significant associations only for liver and CC (Figure 4 and Figure 5). Using the natural-breaks method, we classified the taste index into four ascending levels (Levels 1–4). Both figures exhibited an inverted-U trend: Sour and Sat tastes were positively associated with higher hospitalisation rates for liver and CC at lower taste-index levels (Levels 1–2), whereas this influence diminished progressively as the taste-index level increased (Levels 3–4).

#### Results of interaction detector

Given that we only identified associations between taste and hospitalisations for liver cancer and CC, this study employed the interaction detector to further investigate whether pairwise combinations of the seven taste risk factors would enhance these associations. The results are presented in Table 6. For liver cancer, there were nonlinear enhanced interactions between the following taste pairs: ‘Sweet-Fat’, ‘Umami-Fat’, ‘Salty-Fat’, ‘Fat-Spicy’ and ‘Fat-Pungent.’ For CC, nonlinear enhanced interactions were observed between the taste pairs ‘Sour-Salty’, ‘Sour-Spicy’ and ‘Sour-Pungent’, as well as combinations of ‘Sweet-fat’, ‘Umami-fat’, ‘Salty-fat’, ‘Fat-Spicy’ and ‘Fat-Pungent’.

**Table 6.** Interaction detector results for taste-index pairs and their association with liver and colorectal cancer hospitalisation rates

Cancer	Taste	Sour	Sweet	Umami	Salty	Fat	Spicy	Pungent
Liver cancer	Sour	0·103						
	Sweet	0·187↑	0·051					
	Umami	0·128	0·198↑	0·039				
	Salty	0·172	0·191↑	0·065	0·046			
	Fat	0·186	0·241↑	0·246↑	0·282↑	0·159*		
	Spicy	0·147	0·211↑	0·082	0·092	0·296↑	0·065	
	Pungent	0·172↑	0·192↑	0·09	0·085	0·286↑	0·112	0·035
Colorectal cancer	Sour	0·134*						
	Sweet	0·197↑	0·031					
	Umami	0·175	0·122↑	0·018				
	Salty	0·198↑	0·118↑	0·055	0·03			
	Fat	0·203	0·205↑	0·205↑	0·207↑	0·144*		
	Spicy	0·213↑	0·154↑	0·102↑	0·106↑	0·265↑	0·048	
	Pungent	0·205↑	0·120↑	0·053	0·048	0·211↑	0·115↑	0·032

‘*’ represents *P* < 0·05.

‘↑’ represents that the interaction is non-linear and has enhancement effect.

After combining taste risk factors, the interactive risk value was significantly higher than that of individual tastes. In the case of liver cancer, the highest interactive risk value reached 0·296, approximately twice the single risk value; for CC, it reached 0·265, also about twice the single risk value.

## Discussion

It is widely acknowledged that a healthy diet is considered one of the most important factors for promoting overall health^(33)^. The consumption of different foods can influence population health^(34)^, so the ingredients and seasonings used in their preparation may likewise exhibit a spatial association with individual health outcomes. Drawing on a previous Chinese research, this study adopted a novel approach to examine the association between taste preferences and digestive-system cancer hospitalisation^(16)^. In accordance with the unique characteristics of Chinese cuisine, the taste of each dish is categorised into seven dimensions, namely ‘Sour’, ‘Sweet’, ‘Salty, ‘Umami’, ‘Fat’, ‘Spicy’ and ‘ Pungent’. Because bitter foods are generally considered unpleasant and are not commonly included in diets, we chose to exclude this type of taste from our study. Moreover, as our findings are based on aggregated Internet big data, they serve exclusively for population-level public-health surveillance and are not intended for individualised treatment guidance.

To date, numerous studies in cell biology have demonstrated that taste plays a significant role in human evolution. Taste perception is a crucial factor in human evolution, playing an important role in monitoring food intake and ensuring survival. Taste is not only vital for identifying nutrients and toxins but also influences feeding behaviour and dietary choices. Recent studies have uncovered the relationship between taste perception and genetic genes, revealing that variations in taste receptor genes can lead to differences in taste sensitivity and preferences. For example, the T1R and TAS2R gene families are responsible for detecting sweet, umami and bitter tastes. These genetic variations can influence dietary habits and are associated with health outcomes such as obesity and alcohol consumption^(35,36)^. Moreover, taste receptors are present in tissues outside the oral cavity, such as the gastrointestinal tract and respiratory system, indicating that taste perception is not limited to the mouth but may also play a role in metabolic regulation and immune responses^(37,38)^.

In terms of methodology, since Geodetector only associates digestive system cancers with dietary tastes from a spatial statistical perspective, the lack of selection by Geodetector for other cancers does not imply that they are unrelated to dietary tastes. For instance, in this study, there was no significant association between the taste index and hospitalisation rates for oesophageal and GC. Moreover, the selection of diseases by Geodetector does not necessarily mean that these diseases can be fully explained by one or several specific tastes,because cancer is a complex disease resulting from the interplay of multiple factors and genetic predispositions. It is difficult to attribute its cause to a single factor, such as social behavioural factors^(39)^. A key advantage of Geodetector is its ability to handle nonlinear and non-monotonic relationships, which are commonly present in real-world data but often cannot be adequately captured by traditional correlation coefficients such as Pearson’s or Spearman’s.

Studies have shown that taste preferences are formed early in life and can influence a person’s dietary habits throughout their lifetime. For example, children are innately more attracted to high concentrations of sweet and salty tastes compared with adults, which can lead to excessive intake of sugar and Na, potentially causing health issues such as obesity and hypertension in the future^(40)^. The relationship between taste preferences and cancer risk is not solely determined by genetic factors. Environmental and lifestyle factors, such as exposure to certain foods and dietary patterns, also play a significant role. Dietary customs and food availability can substantially influence taste preferences, which in turn affect health outcomes. For example, individuals who regularly consume diets high in certain tastes, such as salty or spicy foods, may face higher health risks compared with those with more balanced taste profiles^(41,42)^. Additionally, a cohort study of British women found that sensitivity to bitterness is associated with an increased risk of cancer, suggesting that taste preferences may mediate cancer susceptibility by influencing food choices^(43)^.However, we subsequently observed a downward trend in hospitalisation rates as the taste grade continued to increase. We believe this reflects the principle of ‘too much of a good thing can be bad.’ Excessive taste intensity not only affects food choices but may also have direct negative impacts on health. For example, pickled vegetables, which are often prepared with large amounts of salt, may produce nitrites during the pickling process. Nitrites are potential carcinogens, and excessive intake may increase the risk of GC^(44)^.

In terms of taste preferences, patients in Fujian Province tend to Sour and Fat tastes, a preference that may be closely related to cultural and dietary habits. Studies have shown that there are significant differences in preferences for basic tastes among people from different cultural backgrounds. For example, a cross-cultural study compared the preferences for basic tastes among Polish, Tsimane and Hadza populations and found that Tsimane and Polish participants preferred sweetness, while Hadza participants preferred saltiness and sourness^(41)^. Moreover, consuming Sour foods may help alleviate certain gastrointestinal symptoms associated with cancer treatment, such as nausea and loss of appetite. The refreshing and cleansing taste characteristics of sour foods can make them more palatable and easier for patients to accept and consume^(45)^. High-fat foods have been shown to be metabolised by gut microbiota into metabolites such as trimethylamine N-oxide, which is associated with cancer risk^(46)^. Nevertheless, the scientific evidence in this study directly linking taste to cancer incidence remains limited. The interplay between taste perception, dietary choices and health outcomes provides an important framework for understanding potential associations. This framework suggests that Sour and Fat condiments may indirectly influence cancer risk by affecting dietary patterns and nutrient intake, though these hypotheses need to be further validated through rigorous clinical experiments.

We have noted in the results of the interaction detector that both Sour and Fat tastes exhibit active properties. When combined with other tastes, they form compound tastes such as Sour-Salty and Fat-Pungent, which have statistically significant associations with many chronic diseases. For example, pickled vegetables are widely popular due to their salty and sour flavors, yet they are also recognised as a high-risk food for cancer. Moreover, studies have indicated that when fats are cooked together with spices such as garlic and ginger, the spices can generate volatile organic compounds and secondary organic aerosols, which may potentially carry health risks. These examples collectively underscore that the interplay between Sour and Fat tastes and other flavours can amplify their health impacts^(47)^. A study in China that explored the relationship between taste preferences and chronic diseases found that interactions between different tastes can increase the risk of certain diseases. This finding indicates that the combined effects of multiple tastes may have a significant impact on health, highlighting the necessity of understanding taste interactions in dietary interventions^(16)^.

While traditional individual clinical experimental research methods have laid a solid foundation for scientific exploration, they are often accompanied by high costs and limited generalisability. To address this issue, this study proposes an innovative method that involves in-depth analysis of recipes to explore the spatial associations between dietary tastes and the distribution of hospitalisation rates for digestive system cancers, thereby effectively identifying high-risk areas. Although these results have not yet been validated, the value of these exploratory findings lies in providing foundational data for scientific research in the field of public health, reducing research costs, and pointing to new research directions.

There are limitations to our study. First, the conclusions of this study are only applicable to the research area of Fujian Province, as there are significant differences in dietary culture and health environment across different regions. Second, since this study is based on ecological analysis of taste frequency and cancer hospitalisation data, the causality of the results cannot be definitively established. The results imply associations at the community level, which may not be accurate at the individual level and require further validation through individual experimental data. Third, this study may only reflect the taste preferences of the majority of people when dining out and cannot represent everyone. However, it still has a certain degree of representativeness. According to the survey data from iiMedia Research in 2022 (https://www.iimedia.cn/c1061/89770.html), 71·5 % of respondents chose to dine out, indicating that dining out is becoming a trend in China. Although the findings of this study have limitations, its novelty lies in offering a new approach for disease prevention and control departments. By leveraging internet big data, it enables rapid identification of high-risk areas for digestive system cancers.

### Conclusions

Employing Geodetector, we compared the spatial association between seven taste indices distributions and digestive system cancer-hospitalisation rates in Fujian. The study found significant spatial clustering of taste preferences in Fujian Province and an association between Sour and Fat tastes preference and hospitalisation rates for liver and CC, suggesting a dietary taste–cancer link. Furthermore, the co-occurrence of two taste preferences yielded a stronger spatial association with liver and CC hospitalisations than either preference alone.

## Supporting information

Rao et al. supplementary material 1 Rao et al. supplementary material

Rao et al. supplementary material 2 Rao et al. supplementary material

Rao et al. supplementary material 3 Rao et al. supplementary material

Rao et al. supplementary material 4 Rao et al. supplementary material

## Supplementary material

For supplementary material accompanying this paper visit https://doi.org/10.1017/S1368980025101419
